# Supplementary material for: Molecular Detection and Genotyping of Coxiella-Like Endosymbionts in Ticks Collected from Animals and Vegetation in Zambia
Source: Pathogens. 2021 Jun 21;10(6):779. doi: 10.3390/pathogens10060779 (PMC8234379; doi:10.3390/pathogens10060779)
Supplement: Supplementary file 1 [file pathogens-10-00779-s001.zip › pathogens-1261488-SI.pdf]

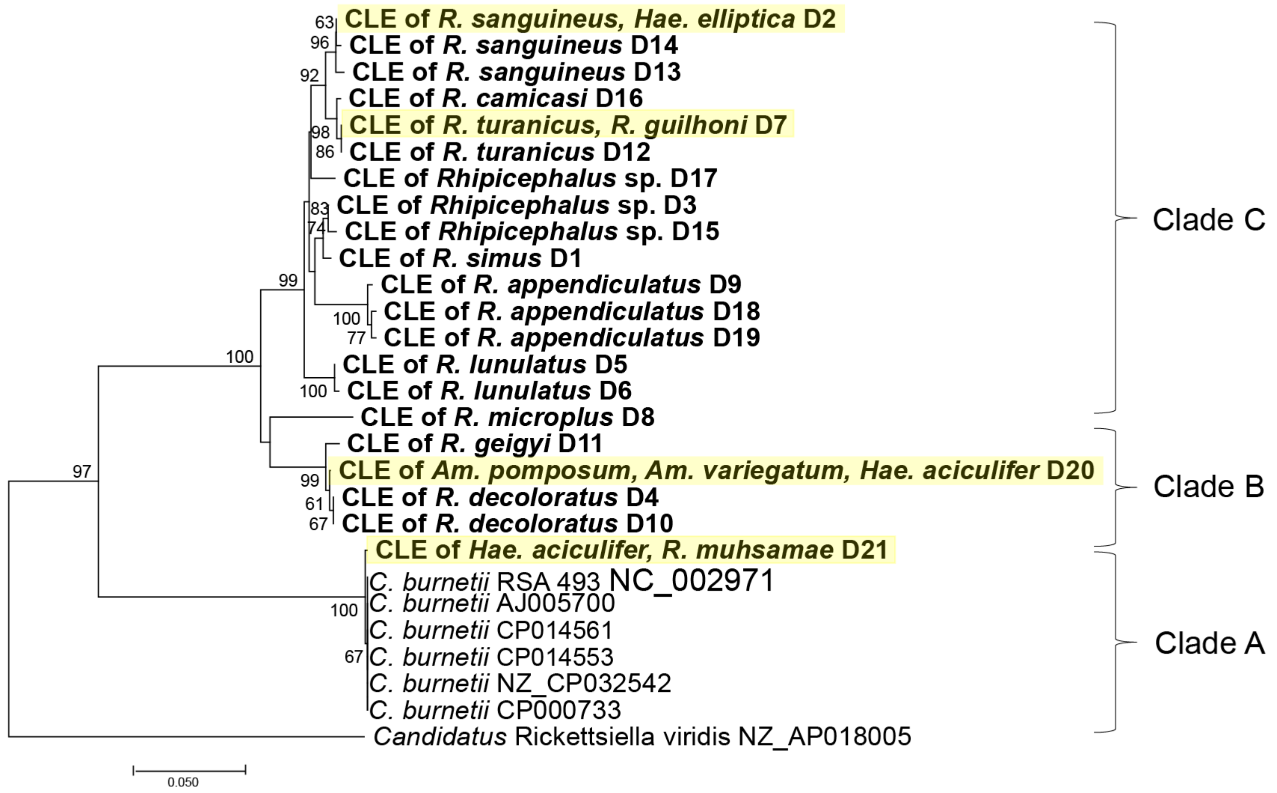

Figure S1. Phylogenetic tree based on the sequences of *dnaK* gene. The tree was constructed using MEGA7 based on the maximum likelihood method, using the Kimura 2-parameter model. All bootstrap values >60 from 1,000 replications are shown on the interior branch nodes. The sequences obtained in this study are in bold. Alleles that were shared by different tick species have been highlighted in yellow. Allele ID is provided next to the tick species name.

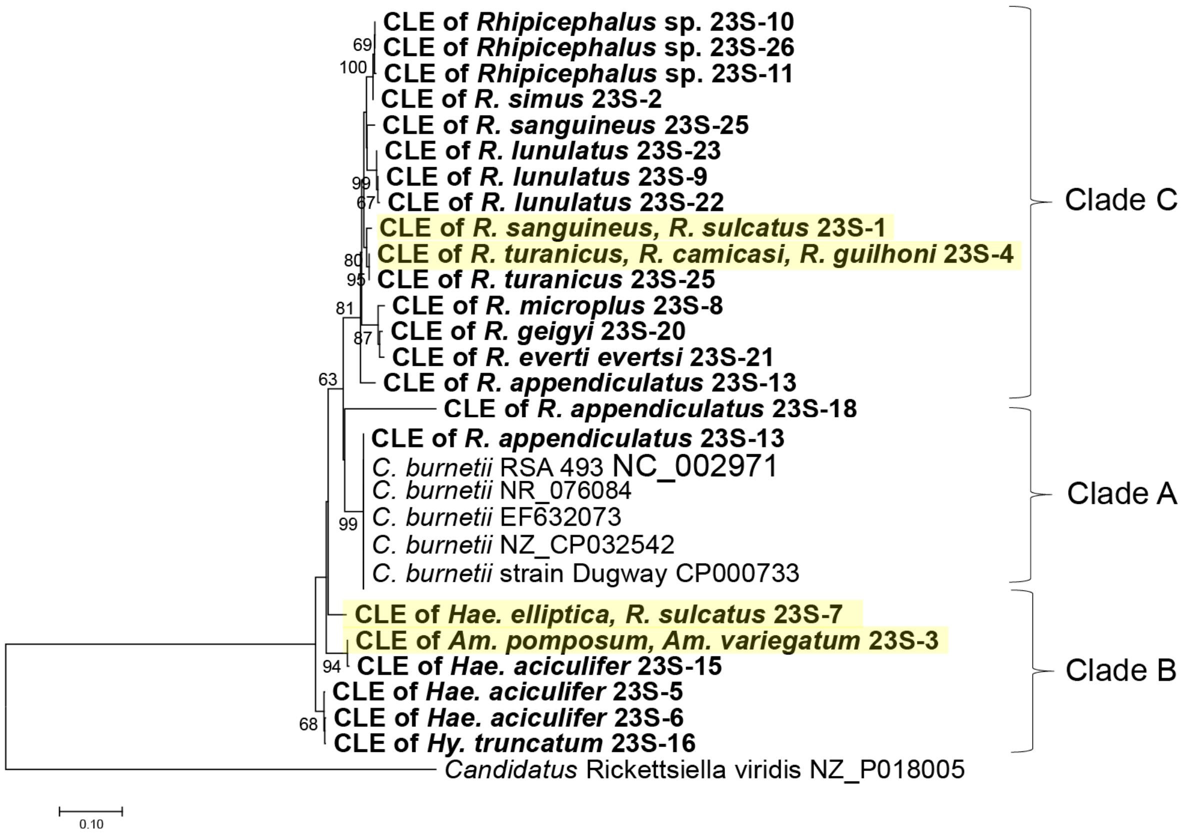

Figure S2. Phylogenetic tree based on the sequences of 23S rRNA gene. The tree was constructed using MEGA7 based on the maximum likelihood method, using the Kimura 2-parameter model. All bootstrap values >60 from 1,000 replications are shown on the interior branch nodes. The sequences obtained in this study are in bold. Alleles that were shared by different tick species have been highlighted in yellow. Allele ID is provided next to the tick species name.

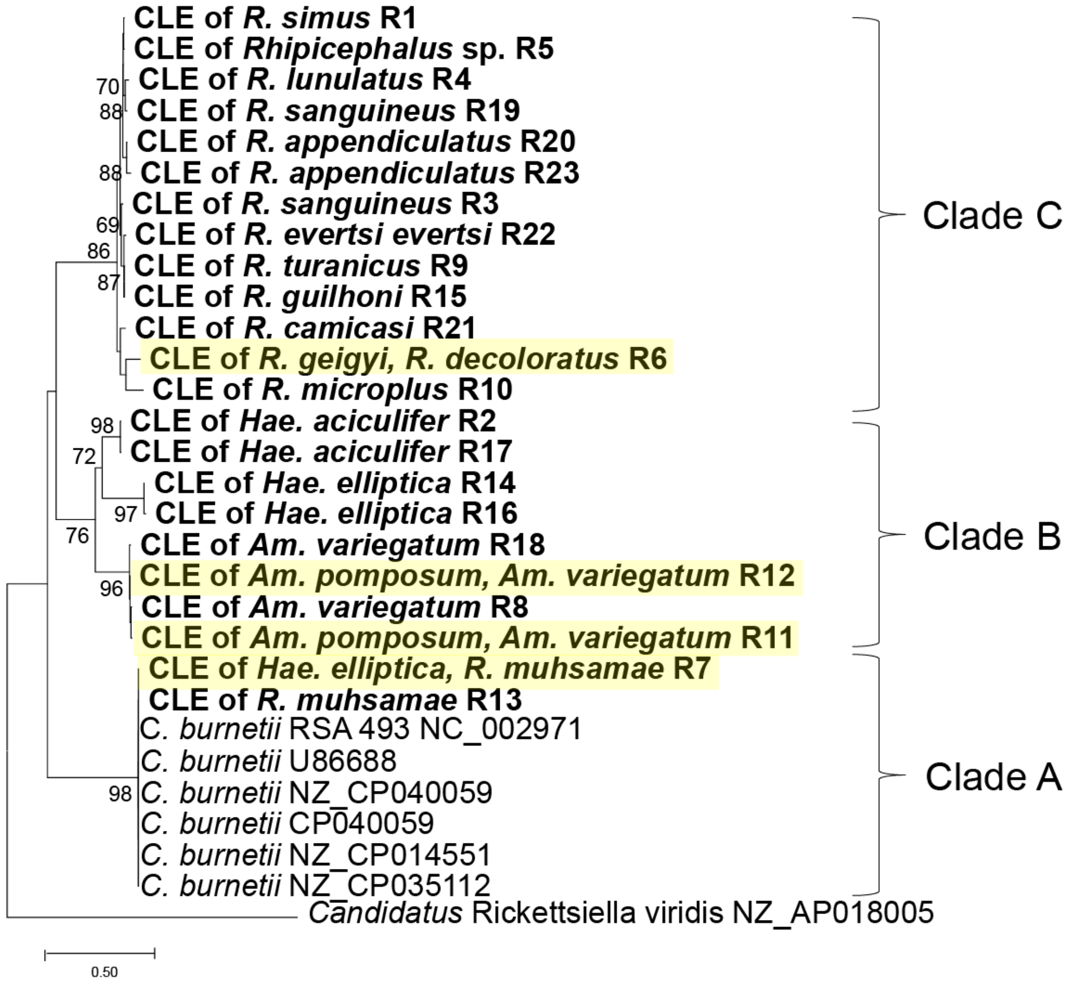

Figure S3. Phylogenetic tree based on the sequences of *rpoB* gene. The tree was constructed using MEGA7 based on t the maximum likelihood method, using the Kimura 2-parameter model. All bootstrap values >60 from 1,000 replications are shown on the interior branch nodes. The sequences obtained in this study are in bold. Alleles that were shared by different tick species have been highlighted in yellow. Allele ID is provided next to the tick species name.
